# The Survival Benefits of Chemotherapy for Undifferentiated Carcinoma With Osteoclast‐Like Giant Cells of Pancreas: A Retrospective Analysis and Individual Participant Data Meta‐Analysis

**DOI:** 10.1002/cam4.70894

**Published:** 2025-05-10

**Authors:** Ouyang Yonghao, Zhi Li, Yi Xiao, Lina Cai, Yannian Liao, Denghuan Lu, Hong Zhu, Rongxi Shen, Xinbo Wang

**Affiliations:** ^1^ Research Institute of General Surgery, Jinling Hospital, Affiliated Hospital of Medical School Nanjing University Nanjing China; ^2^ Department of General Surgery, Jinling Hospital, the First School of Clinical Medicine Southern Medical University Nanjing China

**Keywords:** chemotherapy, survival time, undifferentiated carcinoma with osteoclast‐like giant cells of pancreas (UCOGCP)

## Abstract

**Background and Aim:**

Undifferentiated carcinoma with osteoclast‐like giant cells of pancreas (UCOGCP) is a rare type of pancreatic adenocarcinoma (PDAC). However, the efficacy of chemotherapy in UCOGCP has not been evaluated so far due to the scarcity of cases. This study aims to evaluate the efficacy of chemotherapy in UCOGCP combined with previous individual participant data (IPD) and SEER database data.

**Methods:**

Forty‐nine patients with UCOGCP were enrolled from the Surveillance, Epidemiology, and End Results (SEER) database. Based on whether they had received chemotherapy or not, we divided UCOGCP patients into chemotherapy group (*N* = 32) and non‐chemotherapy group (*N* = 17). The survival time of the chemotherapy group and non‐chemotherapy group was assessed by Kaplan–Meier analysis and Cox analysis. IPD data for UCOGCP were collected in PubMed, Embase, Cochrane, and ScienceDirect. The results based on the SEER database were verified by IPD meta‐analysis.

**Results:**

The Kaplan–Meier analysis indicated that patients who received chemotherapy experienced a longer survival time compared to those who did not (OS: *p* = 0.00061, CSS: *p* = 0.00047). Univariate (OS: HR: 0.31 [0.15, 0.63], *p* = 0.001; CSS: HR: 0.28 [0.13, 0.60], *p* = 0.001) and multivariate (OS: HR: 0.33 [0.14, 0.78], *p* = 0.012; CSS: HR: 0.30 [0.12, 0.73], *p* = 0.008) Cox regression showed that chemotherapy was the independent prognostic protective factor for UCOGCP. IPD meta‐analysis showed that chemotherapy can significantly improve the prognosis of patients who received primary tumor resection (PTR, *p* = 0.0084).

**Conclusion:**

In contrast to not receiving chemotherapy, chemotherapy is effective in prolonging survival in UCOGCP patients with or without PTR. This provides a foundation for the use of UCOGCP chemotherapy.

## 1 Introduction

Undifferentiated pancreatic carcinoma with osteoclast‐like giant cells of pancreas (UCOGCP) first described by Rosai [1], is a rare subtype of pancreatic ductal adenocarcinoma (PDAC), which is essentially characterized by the visualization of osteoclast‐like giant cells in undifferentiated pancreatic cancer tissue [2, 3]. Compared to conventional PDAC, UCOGCP tends to be larger in size and is often associated with polypoid growth, cystic lesions, hemorrhage, and necrosis [3, 4]. With advances in diagnostic techniques, the number of reported UCOGCP cases has increased over the last decade, but it remains a rare disease [5, 6].

Surgical resection is considered the first‐line treatment modality for UCOGCP patients [5, 6]. UCOGCP has the characteristics of being difficult to diagnose at the early stage and easy to relapse [7, 8]. Therefore, the selection of an appropriate adjuvant treatment modality is necessary to reduce recurrence and prolong the survival time of patients. As well as conventional PDAC, chemotherapy is the most commonly used adjuvant modality for UCOGCP and the primary treatment after loss of opportunity for surgery. However, the impact of chemotherapy for UCOGCP remains controversial [5, 6, 9]. Some UCOGCP patients have a better prognosis after chemotherapy [10, 11], but the opposite occurs in some patients [12, 13, 14]. Due to the rare number of cases, very few studies in the past can explore the efficacy of chemotherapy.

The Surveillance, Epidemiology, and End Results (SEER) is a clinical database that collects cancer incidence, prevalence, and survival data from the United States (US) Cancer Registry, which covers approximately 34.6% of the US population [15]. Individual participant data (IPD) meta‐analysis collects, examines, and reanalyzes the raw data for each participant in each study. It is better to assess the integrity of the study compared to conventional meta‐analysis [16]. Therefore, this study combined the SEER database and IPD meta‐analysis of the impact of chemotherapy on the prognosis of UCOGCP patients.

## 2 Materials and Methods

### 2.1 Data Collection

**FIGURE 1:**
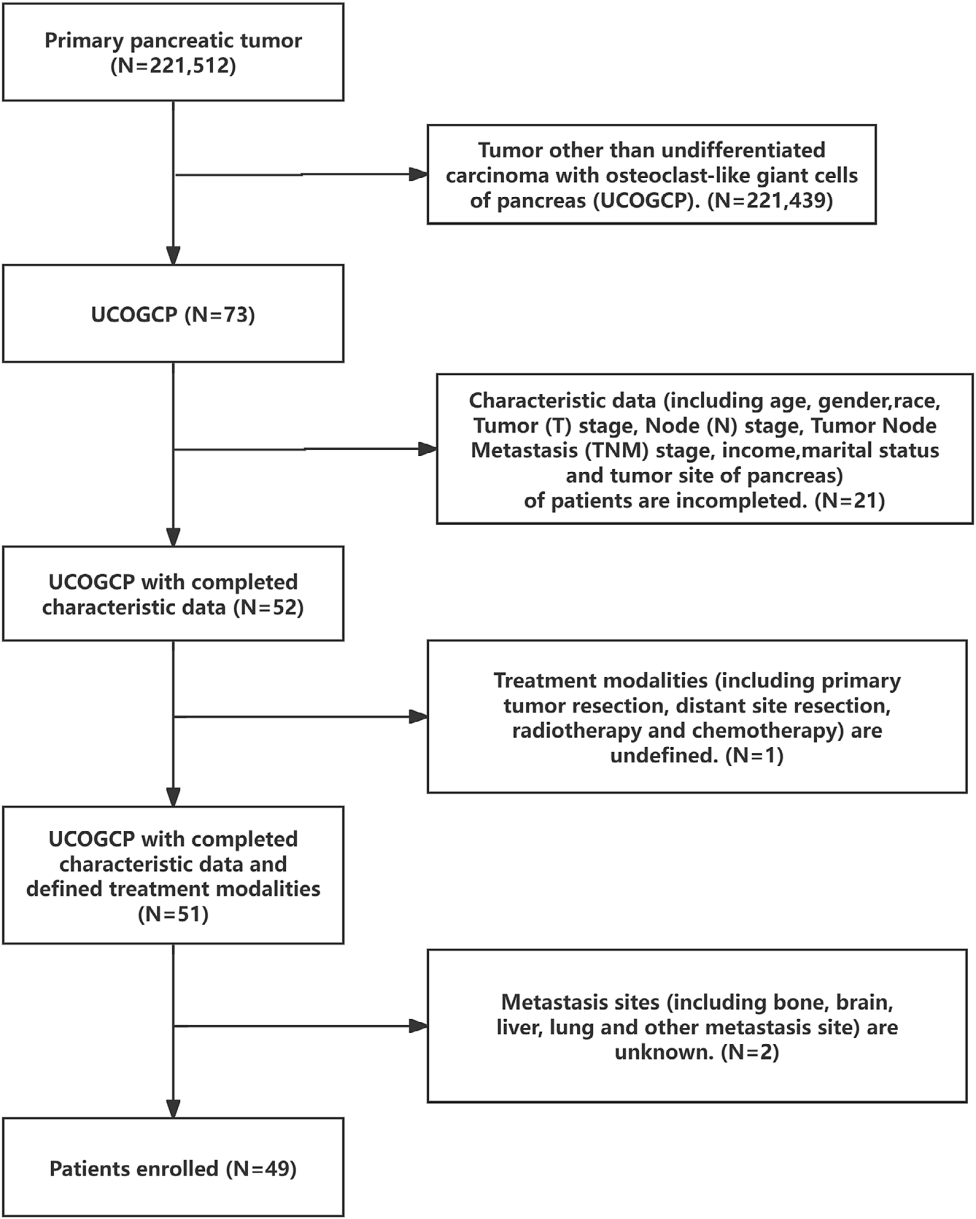
Flowchart of the study participants.

We retrieved the UCOGCP (ICD‐O‐3: 8035/3) data from the SEER database for the years 2000–2020 using SEER*Stat version 8.4.2. Subsequently, we selected the subjects for this study by applying the following exclusion criteria (Figure 1): (1) Inadequate data on patient characteristics, including age, gender, race, stage T, stage N, stage tumor node metastasis (TNM), income, marital status, and tumor site of the pancreas; (2) Unclear treatment modalities, including primary tumor resection (PTR), distant site resection, radiotherapy, and chemotherapy; (3) Indeterminate metastasis sites, such as bone, brain, liver, lung, and other metastasis sites. The inclusion criteria were: availability of complete patient characteristics, treatment modalities, and metastasis sites.

### 2.2 IPD Meta‐Analysis

Following the Preferred Reporting Items for Systematic Reviews and Meta‐Analyses (PRISMA) and Meta‐analysis of Observational Studies in Epidemiology guidelines [16]. Two investigators used the search strategy [(“undifferentiated” or “anaplastic”) and “osteoclast” and “giant” (“pancreas” or “pancreatic”)] to search literature from PubMed, Embase, Cochrane, and ScienceDirect. The results were cross‐checked. Any controversy was solved by a third investigator.

**FIGURE 2:**
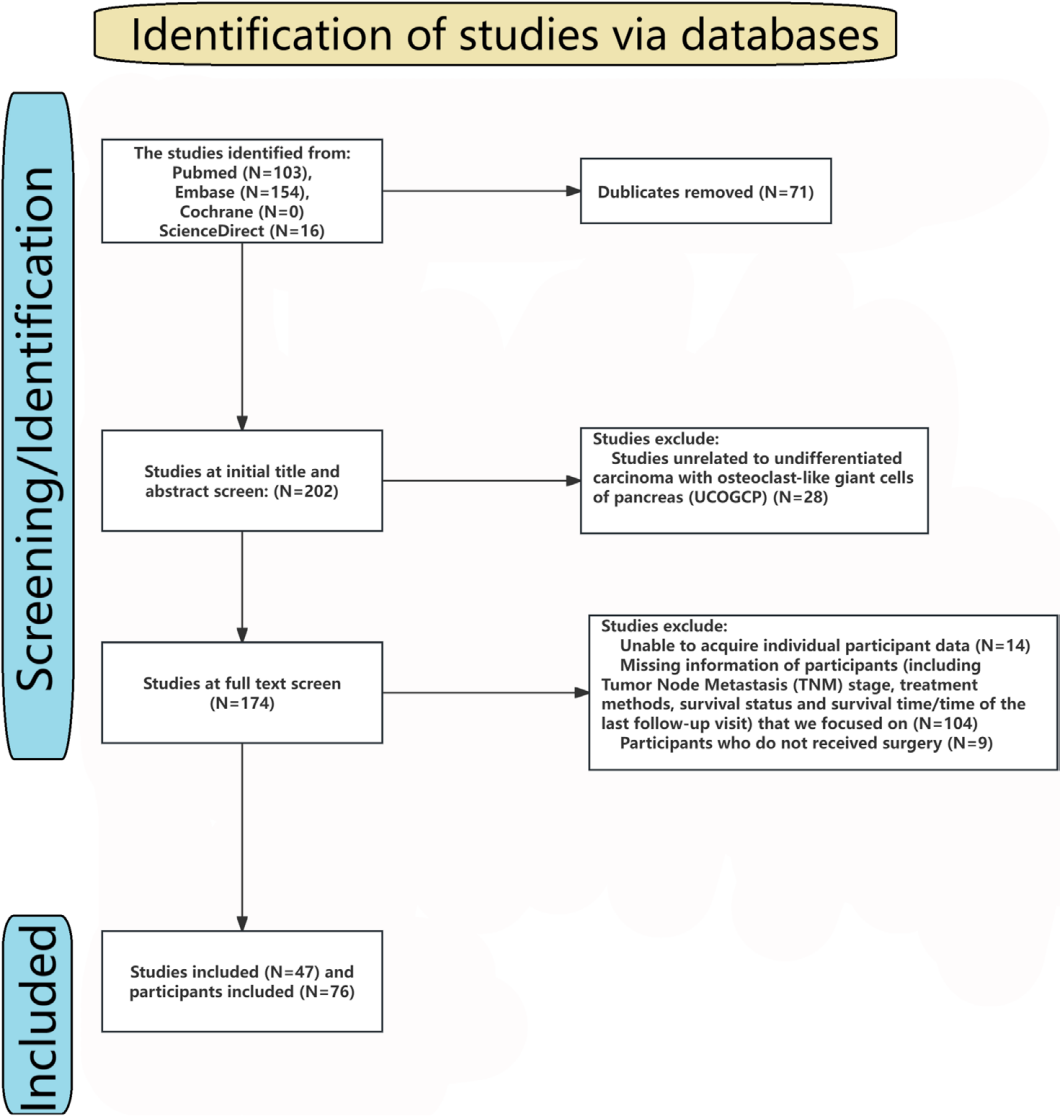
The flow chart of individual participant data (IPD) meta‐analysis.

In this IPD meta‐analysis, we focused on the TNM stage, treatment methods (including surgery, radiotherapy, and chemotherapy), survival status, and survival time or time of the last follow‐up visit. Any literature lacking these data will be excluded. In addition, any studies involving children and animals will also be excluded. Meta‐analysis and review also exclude from the IPD meta‐analysis. The flowchart and the included literature are shown in Table S1 and Figure 2.

### 2.3 Statistical Analysis

Statistical analyses of this study were performed in packages of R language 4.12, including rms, survival, ggplot2, and survminer. *p* < 0.05 was considered statistically significant. Patient characteristics were described using chi‐squared analysis. The Kaplan–Meier curve was used to estimate survival time, and the log‐rank test was used to analyze the differences between different groups. Cox regression was used to screen for the independent prognostic factors.

## 3 Results

### 3.1 Patients' Characteristics

A total of 49 patients with UCOGCP were included in this study (Table 1). The minimum age was 35 years and the maximum age was greater than 85 years. These patients were diagnosed from 2005 to 2020, including 15 (30.61%) patients of the non‐chemotherapy group and 34 (69.39%) patients of the chemotherapy group. None of the patients underwent resection of distant metastasis sites or had concomitant brain or lung metastasis. Patients ≥ 60 years of age constituted the majority of the study subjects. Of the patients with UCOGCP included in our study, 32 (65.31%) patients were female, while 17 (34.69%) patients were male. Half of the patients with UCOGCP had tumors occurring in the head of the pancreas. The majority (67.35%) of patients underwent PTR. The chemotherapy group had a higher proportion of married patients than the non‐chemotherapy group (*p* < 0.05), and no significant difference was observed between the non‐chemotherapy group and chemotherapy group for other characteristics (*p* > 0.05).

**TABLE 1:** The baseline and demographic characteristics of different subgroups.

Characteristic	All (*N* = 49)	Non‐chemotherapy group (*N* = 17)	Chemotherapy group (*N* = 32)	*χ* ^2^	*p*
Age				0.009	0.924
< 60	14 (28.57%)	5 (29.41%)	9 (28.12%)		
≥ 60	35 (71.43%)	12 (70.59%)	23 (71.88%)		
Gender				1.756	0.185
Female	32 (65.31%)	9 (52.94%)	23 (71.88%)		
Male	17 (34.69%)	8 (47.06%)	9 (28.12%)		
Race				0.585	0.746
White	41 (83.68%)	14 (82.36%)	27 (84.38%)		
Black	4 (8.16%)	2 (11.76%)	2 (6.24%)		
Other	4 (8.16%)	1 (5.88%)	3 (9.38%)		
Site				2.206	0.332
Head	26 (53.06%)	7 (41.18%)	19 (59.38%)		
Body and tail	19 (38.78%)	9 (52.94%)	10 (31.24%)		
Overlapping	4 (8.16%)	1 (5.88%)	3 (9.38%)		
Stage T				0.082	0.774
T1–T2	16 (32.65%)	6 (35.29%)	10 (31.25%)		
T3–T4	33 (67.35%)	11 (64.71%)	22 (68.75%)		
Stage N				0.156	0.693
N0	39 (79.59%)	13 (76.47%)	26 (81.25%)		
N1	10 (20.41%)	4 (23.53%)	6 (18.75%)		
Liver metastasis				2.467	0.116
No	38 (77.55%)	11 (64.71%)	27 (84.37%)		
Yes	11 (22.45%)	6 (35.29%)	5 (15.63%)		
Other metastasis				3.337	0.067
No	46 (93.87%)	14 (82.35%)	32 (100.00%)		
Yes	3 (6.06%)	3 (17.65%)	0 (0.00%)		
Stage TNM				1.058	0.589
I–II	36 (73.47%)	11 (64.71%)	25 (78.13%)		
III–IV	13 (26.53%)	6 (35.29%)	7 (21.87%)		
PTR				0.082	0.774
No	16 (32.65%)	6 (35.29%)	10 (31.25%)		
Yes	33 (67.35%)	11 (64.71)	22 (68.75%)		
Radiotherapy				4.353	0.037
No	34 (69.39%)	15 (88.24%)	19 (59.37%)		
Yes	15 (30.61%)	2 (11.76%)	13 (40.63%)		
Income				3.554	0.169
< 55,000	10 (20.41%)	6 (35.29%)	4 (12.50%)		
55,000–74,999	21 (42.86%)	6 (35.29%)	15 (46.87%)		
≥ 75,000	18 (36.73%)	5 (29.42%)	13 (40.63%)		
Marital status				4.864	0.027
No	24 (48.98%)	12 (70.59%)	12 (37.50%)		
Yes	25 (51.02%)	5 (29.41%)	20 (62.50%)		

Abbreviations: Other metastasis, other than bone, brain, liver, and lung; PTR, primary tumor resection; Stage N, stage node; Stage T, stage tumor; Stage TNM, stage tumor node metastasis.

### 3.2 Kaplan–Meier Survival Analysis

**FIGURE 3:**
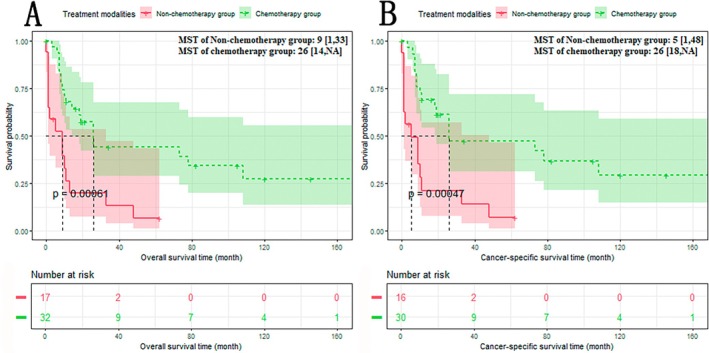
Overall survival (OS) (A) and cancer‐specific survival (CSS) (B) of non‐chemotherapy group and chemotherapy group. A: The OS of the chemotherapy group was significantly longer than that of the non‐chemotherapy group; B: The CSS of the chemotherapy group was significantly longer than that of the non‐chemotherapy group.

We compared the median survival time (MST) of overall survival (OS) and cancer‐specific survival (CSS) of the non‐chemotherapy group and chemotherapy group. The results showed that compared with the non‐chemotherapy group, the prognosis of the chemotherapy group was significantly improved (Figure 3A,B): The non‐chemotherapy group versus the chemotherapy group: OS: 9 versus 26 months, *p* < 0.05; CSS: 5 versus 26 months, *p* < 0.05.

### 3.3 Cox Regression Analysis

**FIGURE 4:**
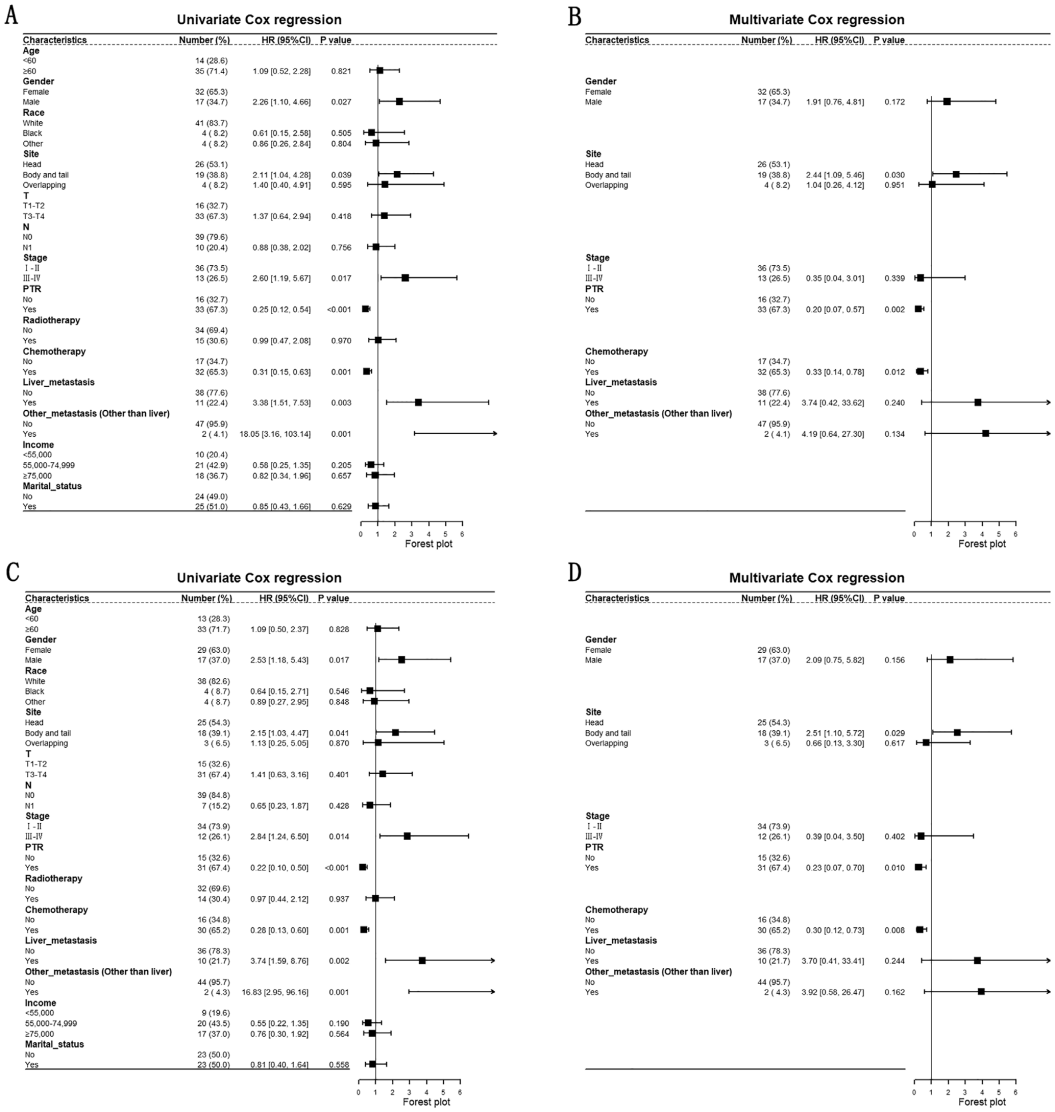
Univariate Cox regression of overall survival (OS) (A) and cancer‐specific survival (CSS) (C); Multivariate Cox regression of OS (B) and CSS (D). A: Gender, site, stage, primary tumor resection (PTR), chemotherapy, liver metastasis, other metastasis (other than liver) are the prognostic factors for OS; B: Site, PTR, chemotherapy are the independent prognostic factors for OS; C: Gender, site, stage, PTR, chemotherapy, liver metastasis, other metastasis (other than liver) are the prognostic factors for CSS; D: Site, PTR, chemotherapy are the independent prognostic factors for CSS.

Subsequently, we explored the independent prognostic factors of UCOGCP by univariate and multivariate Cox regression. The results of univariate Cox regression showed that male (vs. Female, OS: HR: 2.26 [1.10–4.66], *p* = 0.027; CSS: HR: 2.53 [1.18–5.43], *p* = 0.017), body and tail (vs. Head, OS: HR: 2.11 [1.04–4.28], *p* = 0.039; CSS: HR: 2.15 [1.03–4.47], *p* = 0.041), Stage III–IV (vs. Stage I–II, OS: HR: 2.60 [1.19–5.67], *p* = 0.017; CSS: HR: 2.84 [1.24–6.50], *p* = 0.014), PTR yes (vs. no, OS: HR: 0.25 [0.12–0.54], *p* < 0.001; CSS: HR: 0.22 [0.10–0.50], *p* < 0.001), chemotherapy yes (vs. no, OS: HR: 0.35 [0.17–0.73], *p* = 0.005; CSS: HR: 0.33 [0.15–0.70], *p* = 0.004), liver metastasis yes (vs. no, OS: HR: 3.38 [1.51–7.53], *p* = 0.003; CSS: HR: 3.74 [1.59–8.76], *p* = 0.002) and other metastasis (vs. no, OS: HR: 18.05 [3.16–103.14], *p* = 0.001; CSS: HR: 16.83 [2.95–96.16], *p* = 0.001) were prognostic factors for UCOGCP (Figure 4A,C). Male (vs. female), Body and tail (vs. head), Stage III–IV (vs. Stage I–II), liver metastasis yes (vs. no), other metastasis (vs. no) were identified as the prognostic risk factors for UCOGCP. PTR yes (vs. no), chemotherapy yes (vs. no) were recognized as the prognostic protective factors. Consequently, we introduced these prognostic factors (including gender, tumor site of pancreas, TNM stage, liver metastasis status, other metastasis status, with/without PTR, with/without chemotherapy. All of the variables were not adjusted) to multivariate Cox regression. The results showed that body and tail (vs. head, OS: HR: 2.47 [1.10–5.55], *p* = 0.029; CSS: HR: 2.49 [1.10–5.66], *p* = 0.029), PTR yes (vs. no, OS: HR: 0.25 [0.09–0.67], *p* = 0.006; CSS: HR: 0.30 [0.10–0.90], *p* = 0.032), chemotherapy yes (vs. no, OS: HR: 0.39 [0.17–0.93], *p* = 0.034; CSS: HR: 0.32 [0.13–0.81], *p* = 0.016) were independent prognostic factors (Figure 4B,D). Body and tail (vs. head) was the independent prognostic risk factor. PTR yes (vs. no) and chemotherapy yes (vs. no) were the independent prognostic protective factors.

### 3.4 Subgroup Analysis

Due to PTR, the body and tail of the pancreas were the independent prognostic factors. We performed the subgroup analysis based on PTR and the site of the pancreas.

**FIGURE 5:**
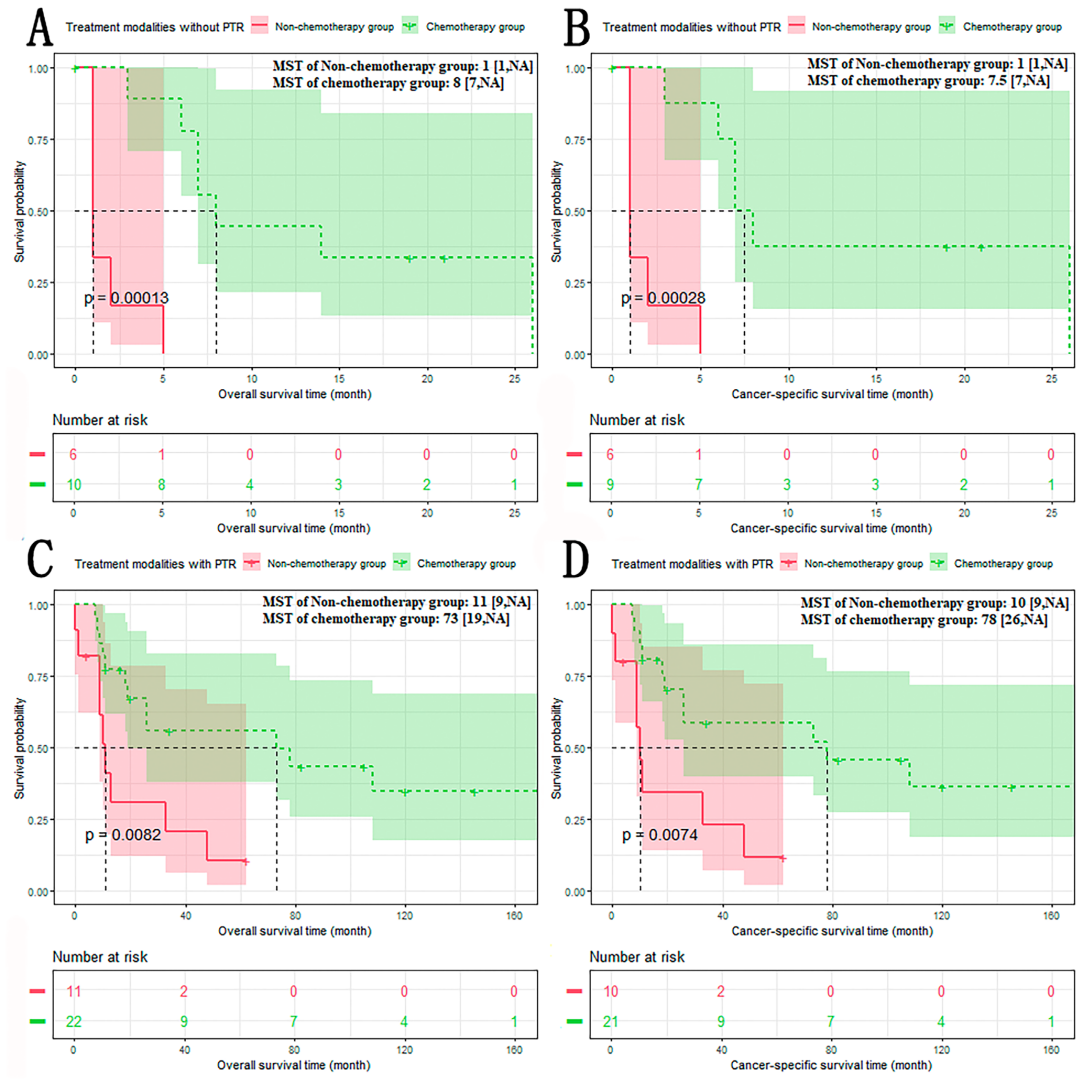
Overall survival (OS)/cancer‐specific survival (CSS) of non‐chemotherapy group and chemotherapy group in non‐primary tumor resection (Non‐PTR) subgroup (A/B) and primary tumor resection (PTR) subgroup (C/D). A: In the Non‐PTR subgroup, the OS of the chemotherapy group was significantly longer than that of the non‐chemotherapy group; B: In the Non‐PTR subgroup, the CSS of the chemotherapy group was significantly longer than that of the non‐chemotherapy group; C: In the PTR subgroup, the OS of the chemotherapy group was significantly longer than that of the non‐chemotherapy group; D: In the PTR subgroup, the CSS of the chemotherapy group was significantly longer than that of the non‐chemotherapy group.

The results of subgroup analysis based on PTR showed that the chemotherapy group had a better prognosis than the non‐chemotherapy group, regardless of whether patients received PTR or not (Figure 5A–D): The non‐chemotherapy group versus the chemotherapy group (treatment modalities with PTR): OS: 11 versus 73 months, *p* < 0.001; CSS: 10 versus 78 months, *p* < 0.001. The non‐chemotherapy group versus the chemotherapy group (treatment modalities without PTR): OS: 1 versus 8 months, *p* < 0.01; CSS: 1 versus 7.5 months, *p* < 0.01.

**FIGURE 6:**
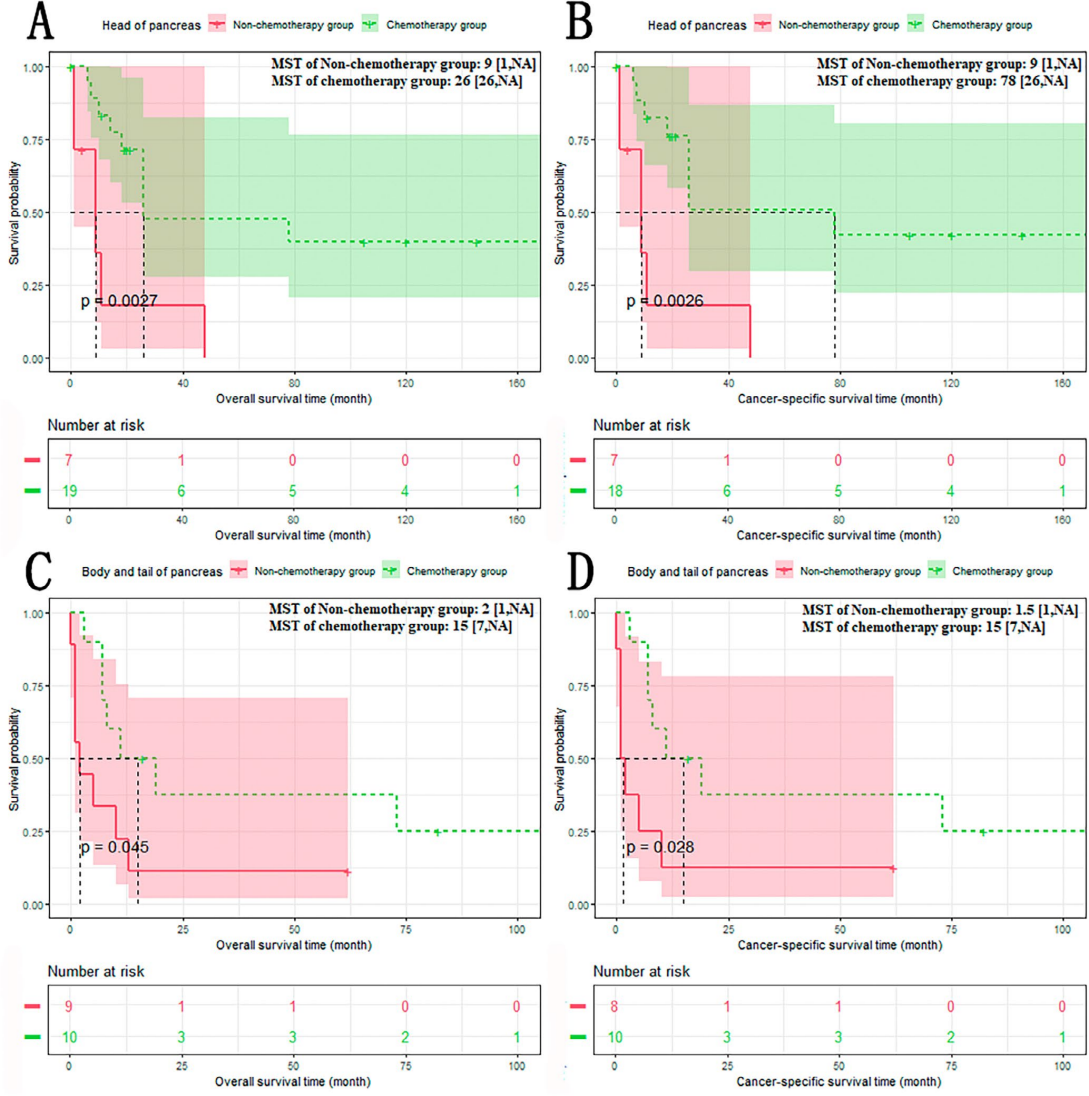
Overall survival (OS)/cancer‐specific survival (CSS) of non‐chemotherapy group and chemotherapy group in head of pancreas subgroup (A/B) and body and tail of pancreas subgroup (C/D). A: In head of pancreas subgroup, OS of chemotherapy group was significantly longer than that of non‐chemotherapy group; B: In head of pancreas subgroup, CSS of chemotherapy group was significantly longer than that of non‐chemotherapy group; C: In body and tail of pancreas subgroup, OS of chemotherapy group was significantly longer than that of non‐chemotherapy group; D: In body and tail of pancreas subgroup, CSS of chemotherapy group was significantly longer than that of non‐chemotherapy group.

The results of subgroup analysis based on site of pancreas showed that the chemotherapy group had a better prognosis than the non‐chemotherapy group, whether the tumor originated from the head, the body, or the tail of the pancreas (Figure 6A–D): The non‐chemotherapy group versus The chemotherapy group (head of pancreas): OS: 9 versus 26 months, *p* < 0.01; CSS: 9 versus 78 months, *p* < 0.01. The non‐chemotherapy group versus the chemotherapy group (body and tail of pancreas): OS: 2 versus 15 months, *p* < 0.05; CSS: 1.5 versus 15 months, *p* < 0.05.

### 3.5 IPD Meta‐Analysis

The baseline information of UCOGCP patients in the meta‐dataset is shown in Table 2. All patients were diagnosed as UCOGCP based on postoperative pathology. All of the 75 patients (from 46 studies) underwent surgical treatment. About half of the patients received additional chemotherapy (neoadjuvant chemotherapy: 5, adjuvant chemotherapy: 29, neoadjuvant combined with adjuvant chemotherapy: 2). There was no statistical difference between the non‐chemotherapy group and chemotherapy group characteristics.

**TABLE 2:** Characteristics of different subgroups in meta‐dataset.

Characteristic	All (*N* = 75)	Non‐chemotherapy group (*N* = 39)	Chemotherapy group (*N* = 36)	*χ* ^2^	*p*
Age				0.900	0.343
< 60	19 (25.33%)	8 (20.51%)	11 (30.56%)		
≥ 60	36 (48.00%)	20 (51.28%)	16 (44.44%)		
Unknown	20 (26.67%)	11 (28.21%)	9 (25.00%)		
Gender				0.072	0.789
Female	27 (36.00%)	13 (33.33%)	14 (38.89%)		
Male	29 (38.67%)	15 (38.46%)	14 (38.89%)		
Unknown	19 (25.33%)	11 (28.21%)	8 (22.22%)		
Race				Fisher test	1.000
White	3 (4.00%)	2 (5.13%)	1 (2.78%)		
Asian	3 (4.00%)	1 (2.56%)	2 (5.55%)		
Unknown	69 (92.00%)	36 (92.31%)	33 (91.67%)		
Site				2.500	0.286
Head	18 (24.00%)	7 (17.95%)	11 (30.56%)		
Body and tail	24 (32.00%)	8 (20.51%)	16 (44.44%)		
Overlapping	4 (5.33%)	3 (7.69%)	1 (2.78%)		
Unknown	29 (38.67%)	21 (53.85%)	8 (22.22%)		
Stage T				0.100	0.752
T1–T2	34 (45.33%)	17 (43.59%)	17 (47.22%)		
T3–T4	41 (54.67%)	22 (56.41%)	19 (52.78%)		
Stage N				0.066	0.797
N0	51 (68.00%)	26 (66.67%)	25 (69.44%)		
N1	24 (32.00%)	13 (33.33%)	11 (30.56%)		
Metastasis				0.005	0.944
No	72 (96.00%)	38 (97.44%)	34 (94.44%)		
Yes	3 (4.00%)	1 (2.56%)	2 (5.56%)		
Stage TNM				0.214	0.644
I–II	60 (80.00%)	32 (82.05%)	28 (77.78%)		
III–IV	15 (20.00%)	7 (17.95%)	8 (22.22%)		
Radiotherapy				1.039	0.308
No	70 (93.33%)	38 (97.44%)	32 (88.89%)		
Yes	5 (6.67%)	1 (2.56%)	4 (11.11%)		

Abbreviations: Stage N, stage node; Stage T, stage tumor; Stage TNM, stage tumor node metastasis.

**FIGURE 7:**
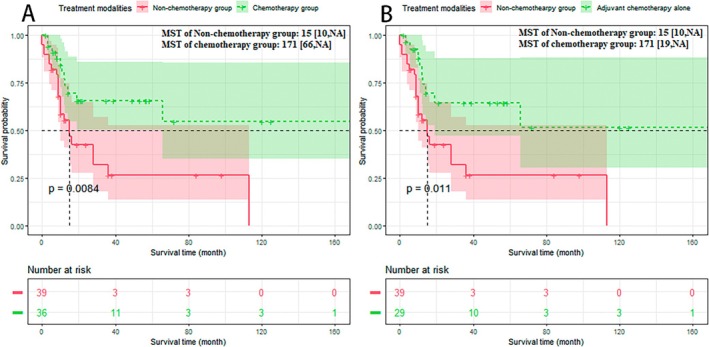
Survival time of non‐chemotherapy group and chemotherapy group in meta‐dataset (A); Survival time of non‐chemotherapy group and adjuvant chemotherapy alone in meta‐dataset (B). A: The survival time of the chemotherapy group was significantly longer than the non‐chemotherapy group; B: The survival time of the adjuvant chemotherapy alone group was significantly longer than the non‐chemotherapy group.

The Kaplan–Meier analysis was performed based on the meta‐dataset (Figure 7A): The non‐chemotherapy group versus the chemotherapy group: 15 versus 171 months, *p* < 0.01. To eliminate discrepancies between neoadjuvant and adjuvant chemotherapy, a Kaplan–Meier analysis was also performed on patients receiving adjuvant chemotherapy alone (Figure 7B): The non‐chemotherapy group versus the chemotherapy group: 15 versus 171 months, *p* < 0.05.

**FIGURE 8:**
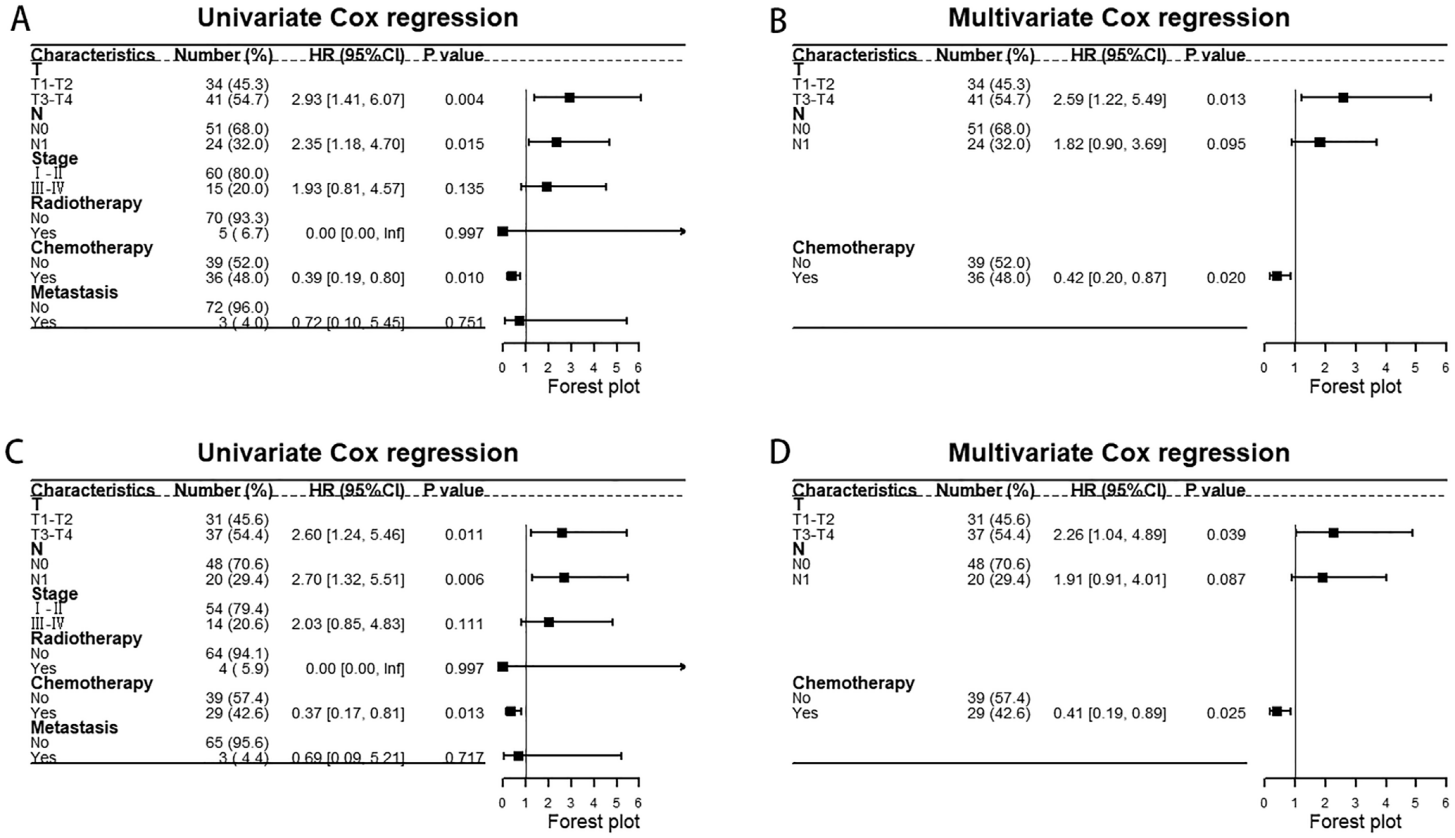
Univariate Cox regression (A) and multivariate Cox regression (B) in the meta‐dataset; Univariate Cox regression (C) and multivariate Cox regression (D) in the meta‐dataset after excluding patients who received neoadjuvant chemotherapy. A: Stage T, stage N, chemotherapy are the prognostic factors for survival time; B: Stage T, chemotherapy is the independent prognostic factor; C: Stage T, stage N, adjuvant chemotherapy alone are the prognostic factors for survival time; D: Stage T, adjuvant chemotherapy alone is the independent prognostic factor.

The Cox regression results based on SEER showed that male, advanced stage, head of pancreas, PTR, and metastasis were the prognostic factors for UCOGCP. After excluding patients which were with unknown tumor site, age, or received neoadjuvant chemotherapy, we further validated this by univariate and multivariate Cox regression based on the metadata set (Figure 8A,B). The results of univariate Cox regression showed that T3–T4 (vs. T1–T2, HR: 2.93 [1.41–6.07], *p* = 0.004), N1 (vs. N0, HR: 2.35 [1.18–4.70], *p* = 0.015), chemotherapy yes (vs. no, HR: 0.39 [0.19–0.80], *p* = 0.010) were prognostic factors for UCOGCP. The results of multivariate Cox regression showed that T3–T4 (vs. T1–T2, HR: 2.59 [1.22–5.49], *p* = 0.013), chemotherapy yes (vs. no, HR: 0.42 [0.20–0.87], *p* = 0.020) were independent prognostic factors. Similarly, we excluded patients who received neoadjuvant chemotherapy and performed Cox regression (Figure 8C,D). The results of univariate Cox regression showed that T3–T4 (vs. T1–T2, HR: 2.60 [1.24–5.46], *p* = 0.011), N1 (vs. N0, HR: 2.70 [1.32–5.51], *p* = 0.006), chemotherapy yes (vs. no, HR: 0.37 [0.17–0.81], *p* = 0.013) were prognostic factors for UCOGCP. The results of multivariate Cox regression showed that T3–T4 (vs. T1–T2, HR: 2.26 [1.04–4.89], *p* = 0.039), chemotherapy yes (vs. no, HR: 0.41 [0.19–0.89], *p* = 0.025) were independent prognostic factors.

## 4 Discussion

UCOGCP is a rare subtype of PDAC and many studies believe that the prognosis of UCOGCP is better than that of [2, 3, 17]. Nevertheless, our study found that the prognosis of UCOGCP was still poor (the MST of SEER dataset: 14 months, the MST of meta‐dataset: 28 months). Therefore, there is a need to explore treatment modalities of UCOGCP.

Surgery is considered the first line modality for UCOGCP, while the effects of chemotherapy on UCOGCP remain unclear due to the number of cases [17]. Therefore, treatment modalities for conventional PDAC are generally also applied in UCOGCP. Surgery (based on site and involved range of tumor) and chemotherapy (mainly including gemcitabine, FOLFIRINOX, AG, and S‐1 regimen) are the main treatment options for UCOGCP. In addition, radiotherapy and immunotherapy are also alternative treatment modalities for UCOGCP [11, 18, 19, 20, 21]. In contrast to the conventional PDAC, UCOGCP is often accompanied by PD‐L1 expression, and it is associated with a poor prognosis [21, 22]. Although Hrudka et al. recommend immune checkpoint immunotherapy for patients with advanced PD‐L1 positive UCOGC [21]. However, the efficacy of the immune checkpoint is still controversial. In the cases of Besaw et al., immune checkpoint inhibitors showed significant efficacy in the primary site of UCOGCP and metastasis [23]. However, the case report of Obayashi et al. suggests that immune checkpoint inhibitors have limited effects on primary tumors but may have antitumor effects on UCOGC lung metastatic lesions [24]. Chemotherapy, as the most common adjuvant and neoadjuvant modality of UCOGCP, has only been used in case reports due to the limitation of the number of cases. Kobayashi et al. reported a patient with UCOGCP who received two surgeries and adjuvant chemotherapy with gemcitabine and eventually survived for 66 months after the first surgery [25]. Yazawa et al. reported a case receiving S‐1, FOLFIRINOX, and AG after PD surgery but survived only 1 year after surgery [26]. Hrudka et al. reported that 5/13 UCOGCP patients (one of whom had a mixed tumor) received postoperative adjuvant chemotherapy [14]. One of the five patients survived 171 months after surgery, with the longest survival time among patients with follow‐up outcomes in previous studies collected by us [14]. Mattiolo et al. reported 4/16 UCOGCP patients (of which one had unknown survival time and vital status) who received neoadjuvant chemotherapy, and these three patients (excluding patients with unknown survival time and vital status) had survival of over 1 year but no deaths were observed [27]. In the study of Matsubayashi et al., a 73‐year‐old male with UCOGCP received preoperative neoadjuvant S‐1 and postoperative adjuvant gemcitabine but survived only 8 months [28]. The conclusions of these studies are still quite different, so studies with large samples still need to be verified.

We extracted 49 and 75 UCOGCP patients from the SEER database and from previous studies, respectively. We found that UCOGCP occurs mostly in elderly patients, which is reflected in both the SEER dataset (35/49) and the meta‐dataset (36/75) and is consistent with the prevalent features of pancreatic cancer [29]. The analysis based on the SEER dataset showed that patients who received radiotherapy were often receiving concurrent chemotherapy (2/17 vs. 13/32, *p* < 0.05). This is also seen in the meta‐dataset, but the difference is not statistically significant (1/39 vs. 4/36, *p* > 0.05). The analysis based on the SEER dataset showed that married patients were preferred to be exposed to chemotherapy. Baine et al. believe that married patients are more likely to receive social and spiritual support [30]. Previous reports have indicated that unmarried patients tend to have shorter survival times and are more likely to remain untreated for cancer compared to their married counterparts [30, 31, 32].

To observe the efficacy of chemotherapy, we divided the patients into non‐chemotherapy group and chemotherapy group. Kaplan–Meier analysis showed that both OS and CSS were significantly longer in the chemotherapy group than in the non‐chemotherapy group, which is consistent with the results obtained for most of the case reports [5, 10, 11, 20, 27, 28, 33, 34]. Subsequently, univariate and multivariate Cox regression showed that chemotherapy is an independent prognostic protective factor for UCOGCP in both the SEER dataset and the meta‐dataset, which is inconsistent with the results of Han et al. in common pancreatic cancer [35], but is inconsistent with the invasive intraductal papillary‐mucinous carcinoma (another rare subtype of pancreas with which UCOGCP often exists) described by McMillan et al. [14, 36, 37]. This indicates the stability of chemotherapy for UCOGCP efficacy. Univariate and multivariate Cox regression based on the SEER dataset showed that tumor site of pancreas and PTR are also independent prognostic factors for UCOGCP. However, it contradicts the findings of Huang et al. in the context of common pancreatic cancer without regional lymph node metastasis [38]. In a study by Yun et al., it is believed that the site of pancreas is not associated with the prognosis of common pancreatic cancer [35], indicating that adjuvant chemotherapy is able to stably improve the efficacy of PTR. As the tumor site of pancreas and PTR are independent prognostic factors for UCOGCP, we confirmed by subgroup analysis that chemotherapy was able to exert stable efficacy in each PTR subgroup and the site of pancreas subgroup, which provides an alternative therapeutic approach for UCOGCP patients who did not receive chemotherapy and is similar to the conclusion of a previous study in conventional PDAC [39, 40]. Compared with adjuvant chemotherapy, neoadjuvant chemotherapy prefers the patient population with relatively later tumor stage [39, 41], which may result in patients receiving neoadjuvant chemotherapy with a later tumor stage and a worse prognosis than patients receiving adjuvant chemotherapy. In our aggregated meta‐dataset, the patients reported by Matsubayashi et al. (UCOGCP patients with neoadjuvant and adjuvant) and Smith et al. (UCOGCP patients with adjuvant) have a poor prognosis with survival time less than 1 year [13, 28]. About half of UCOGCP patients (4/7) who received neoadjuvant or neoadjuvant combined with adjuvant were with lymph node metastasis in our meta‐dataset. Therefore, to exclude this bias, we performed Kaplan–Meier and Cox regression based on the meta‐dataset after excluding UCOGCP patients who received neoadjuvant chemotherapy or neoadjuvant combined with adjuvant chemotherapy. The results showed that adjuvant chemotherapy could still significantly improve the survival time of UCOGCP patients treated with PTR.

The advantages of this study are: (1) This study is based on a multicenter large‐volume sample database and meta‐dataset, which provided UCOGCP patients for analysis; (2) Univariate and multivariate Cox regression analyses and Kaplan–Meier analyses were performed to compare the effects of chemotherapy on the prognosis of the patients; (3) Based on the results of Cox regression, subgroup analysis was performed to confirm the stability of the effect of chemotherapy for UCOGCP; (4) The chemotherapy effects on the prognosis of UCOGCP patients were verified by IPD meta‐analysis, and results of the IPD meta‐analysis complemented the results from the SEER database.

However, this study had some imperfections: (1) For a retrospective study, such an experiment may be biased; (2) Due to the small number of cases that did not receive PTR in the previous studies, the efficacy of chemotherapy in a population of patients who did not receive PTR could not be verified by IPD meta‐analysis; (3) Due to the limitation of the number of cases, the efficacy of neoadjuvant chemotherapy and different chemotherapy regimens cannot be evaluated by IPD meta‐analysis.

In conclusion, we compared the prognosis of UCOGCP patients who received or did not receive chemotherapy through the SEER database and meta‐dataset. Chemotherapy can improve the survival time for UCOGCP with or without PTR, which provides the rationale for the use of UCOGCP chemotherapy.

## Author Contributions

**Ouyang Yonghao:** software, methodology, visualization, writing – original draft, writing – review and editing, data curation. **Zhi Li:** writing – review and editing, data curation. **Yi Xiao:** writing – review and editing, data curation. **Lina Cai:** writing – review and editing. **Yannian Liao:** writing – review and editing. **Denghuan Lu:** writing – review and editing. **Hong Zhu:** writing – review and editing. **Rongxi Shen:** writing – review and editing. **Xinbo Wang:** writing – review and editing.

## Ethics Statement

The study did not require approval from the ethics review committee.

## Conflicts of Interest

The authors declare no conflicts of interest.

## Supporting information


**Table S1.** The included literature for individual participant data (IPD) meta‐analysis.

## Data Availability

The data that support the findings of this study are available from the corresponding author upon reasonable request.
