# Supplementary material for: The Survival Benefits of Chemotherapy for Undifferentiated Carcinoma With Osteoclast‐Like Giant Cells of Pancreas: A Retrospective Analysis and Individual Participant Data Meta‐Analysis
Source: Cancer Med. 2025 May 10;14(9):e70894. doi: 10.1002/cam4.70894 (PMC12065096; doi:10.1002/cam4.70894)
Supplement: Supplementary file 1 — Table S1. The included literature for individual participant data (IPD) meta‐analysis. [file CAM4-14-e70894-s001.docx]

**Table S1 The included literature for IPD meta-analysis**

| **Author** | **Age** | **Gender** | **Race** | **Site** | **T** | **N** | **M** | **PTR** | **Radiotherapy** | **Chemotherapy** | **Number of cycles** | **Reasons for do not received chemotherapy** | **Other metastasis** | **Survival time** | **Vital status** | **Published date** |
| --- | --- | --- | --- | --- | --- | --- | --- | --- | --- | --- | --- | --- | --- | --- | --- | --- |
| Njoumi et al.[1] | 60 | Female | ~ | Body and tail | 1 | 0 | 0 | SPDP | No | No | ~ | Patient rejects | No | 9 | Alive | 2014 |
| Jo et al.[2] | 67 | Female | ~ | Neck and body | 3 | 1 | 0 | DP | No | No | ~ | ~ | No | 9 | Dead | 2014 |
| Temesgen et al.[3] | 56 | Female | White | Tail | 3 | 1 | 0 | DP | Yes | No | ~ | ~ | No | 6 | Alive | 2014 |
| Jung et al.[4] | 74 | Female | ~ | Body | 2 | 0 | 0 | DP | No | S-1 | 1 | ~ | No | 2 | Alive | 2014 |
| Kobayashi et al.[5] | 37 | Female | ~ | Body and tail | 2 | 0 | 0 | PPPD | No | Gemcitabine | 5 | ~ | No | 66 | Dead | 2015 |
| Gao et al.[6] | 71 | Female | ~ | Body and tail | 3 | 0 | 0 | DP | No | Gemcitabine | 4 cycles, then 1 per year | ~ | No | 120 | Alive | 2015 |
| Chiarelli et al.[7] | 68 | Female | White | Neck and body | 3 | 0 | 0 | TP | ~ | Gemcitabine | ~ | ~ | No | 10 | Dead | 2015 |
| Sunahara et al.[8] | 84 | Female | ~ | Tail | 4 | 1 | 0 | DP | No | S-1 | ~ | ~ | No | 7 | Alive | 2015 |
| Georgiou et al.[9] | 75 | Female | ~ | Head | 4 | 0 | 0 | PD | No | No | ~ | Patient rejects | No | 10 | Dead | 2016 |
| Saito et al.[10] | 61 | Female | ~ | Tail | 3 | 0 | 0 | DP | No | Gemcitabine+S-1 | 50 | ~ | No | 72 | Alive | 2016 |
| Luchini et al.[11] 3ST4 | 85 | Female | ~ | ~ | 3 | 1 | 0 | PD | No | No | ~ | ~ | No | 9 | Dead | 2017 |
| Luchini et al.[11] 3ST5 | 66 | Male | ~ | ~ | 2 | 0 | 0 | PD | No | No | ~ | ~ | No | 8 | Dead | 2017 |
| Luchini et al.[11] 3ST6 | 55 | Female | ~ | ~ | 3 | 0 | 0 | PD | No | No | ~ | ~ | No | 28 | Dead | 2017 |
| Luchini et al.[11] 3ST9 | 72 | Male | ~ | ~ | 2 | 0 | 0 | PD | No | No | ~ | ~ | No | 15 | Dead | 2017 |
| Luchini et al.[11] 3ST10 | 49 | Female | ~ | ~ | 3 | 1 | 0 | PD | No | No | ~ | ~ | No | 0 | Dead | 2017 |
| Luchini et al.[11] 3ST11 | 54 | Male | ~ | ~ | 3 | 1 | 0 | PD | No | No | ~ | ~ | No | 10 | Dead | 2017 |
| Luchini et al.[11] 3ST14 | 68 | Male | ~ | ~ | 3 | 0 | 0 | PD | No | No | ~ | ~ | No | 16 | Dead | 2017 |
| Luchini et al.[11] 3ST15 | 64 | Male | ~ | ~ | 2 | 0 | 0 | DP | No | No | ~ | ~ | No | 36 | Dead | 2017 |
| Luchini et al.[11] 3ST19 | 68 | Male | ~ | ~ | 1 | 0 | 0 | DP | No | No | ~ | ~ | No | 5 | Dead | 2017 |
| Luchini et al.[11] 3ST20 | 64 | Female | ~ | ~ | 3 | 1 | 0 | PD | No | No | ~ | ~ | No | 12 | Dead | 2017 |
| Yazawa et al.[12] | 54 | Male | ~ | Body and tail | 4 | 0 | 0 | DP | No | FOLFIRINOX+AG+S-1 | ~ | ~ | No | 12 | Dead | 2017 |
| Okamuara et al.[13] | 84 | Female | ~ | Body | 3 | 1 | 0 | DP | No | Gemcitabine | 9 | ~ | No | 19 | Dead | 2017 |
| Guo et al.[14] | 65 | Male | Asian | Head | 4 | 0 | 0 | PD | No | No | ~ | Patient rejects | No | 10 | Alive | 2018 |
| Zhang et al.[15] | 57 | Male | ~ | Tail | 3 | 0 | 0 | DP | No | Yes | ~ | ~ | No | 3 | Alive | 2018 |
| Hanayneh et al.[16] | 59 | Female | ~ | Head | 2 | 0 | 0 | PD | No | No | ~ | ~ | No | 24 | Alive | 2019 |
| Sato et al.[17] | 61 | Female | Asian | Head | 2 | 0 | 0 | DP | No | TS-1 | ~ | ~ | No | 35 | Alive | 2019 |
| Matsubayashi et al.[18] | 73 | Male | ~ | Head | 3 | 0 | 0 | PD | No | Gemcitabine+S-1 | ~ | ~ | No | 8 | Dead | 2019 |
| Nehmeh et al.[19] | 77 | Male | White | Tail | 2 | 0 | 0 | DP | No | No | ~ | ~ | No | 19 | Alive | 2019 |
| Shi et al.[20] | 31 | Female | ~ | Tail | 3 | 0 | 0 | DP | No | FOLFIRINOX | ~ | ~ | No | 3 | Alive | 2019 |
| Uemura et al.[21] | 71 | Female | ~ | Head | 4 | 0 | 0 | PD | No | No | ~ | ~ | No | 4 | Dead | 2019 |
| Miyagawa et al.[22] | 53 | Female |  | Head | 1 | 0 | 0 | PPPD+DP | No | Gemcitabine | ~ | ~ | No | 125 | Alive | 2020 |
| Yang et al.[23] | 31 | Male | ~ | Head | 3 | 0 | 0 | PD | No | FOLFIRINOX+AG | ~ | ~ | No | 12.6 | Dead | 2020 |
| Obayashi et al.[24] | 66 | Male | ~ | Tail | 2 | 1 | 1 | DP | No | No | ~ | ~ | Yes | 6 | Alive | 2020 |
| Mattiolo et al.[25] 1 | ~ | ~ | ~ | ~ | 2 | 1 | 0 | 1 | No | No | ~ | ~ | No | 113 | Dead | 2020 |
| Mattiolo et al.[25] 2 | ~ | ~ | ~ | ~ | 3 | 1 | 0 | 1 | No | No | ~ | ~ | No | 9 | Dead | 2020 |
| Mattiolo et al.[25] 3 | ~ | ~ | ~ | ~ | 2 | 0 | 0 | 1 | No | No | ~ | ~ | No | 28 | Dead | 2020 |
| Mattiolo et al.[25] 5 | ~ | ~ | ~ | ~ | 2 | 0 | 0 | 1 | No | Yes | ~ | ~ | No | 72 | Alive | 2020 |
| Mattiolo et al.[25] 6 | ~ | ~ | ~ | ~ | 2 | 0 | 0 | 1 | No | No | ~ | ~ | No | 15 | Dead | 2020 |
| Mattiolo et al.[25] 7 | ~ | ~ | ~ | ~ | 3 | 1 | 0 | 1 | No | No | ~ | ~ | No | 0 | Dead | 2020 |
| Mattiolo et al.[25] 8 | ~ | ~ | ~ | ~ | 2 | 1 | 0 | 1 | No | Yes | ~ | ~ | No | 22 | Alive | 2020 |
| Mattiolo et al.[25] 10 | ~ | ~ | ~ | ~ | 3 | 0 | 0 | 1 | No | No | ~ | ~ | No | 9 | Alive | 2020 |
| Mattiolo et al.[25] 11 | ~ | ~ | ~ | ~ | 3 | 0 | 0 | 1 | No | No | ~ | ~ | No | 10 | Alive | 2020 |
| Mattiolo et al.[25] 12 | ~ | ~ | ~ | ~ | 2 | 1 | 0 | 1 | No | Yes | ~ | ~ | No | 19 | Alive | 2020 |
| Mattiolo et al.[25] 14 | ~ | ~ | ~ | ~ | 3 | 0 | 0 | 1 | No | No | ~ | ~ | No | 12 | Alive | 2020 |
| Mattiolo et al.[25] 16 | ~ | ~ | ~ | ~ | 2 | 0 | 0 | 1 | No | No | ~ | ~ | No | 12 | Alive | 2020 |
| Cai et al.[26] | 54 | Male | ~ | Body and tail | 3 | 0 | 0 | STP | No | No | ~ | Patient rejects | No | 84 | Alive | 2020 |
| Yamamura et al.[27] | 68 | Female | ~ | Head | 1 | 0 | 0 | PPPD | No | S-1 | ~ | ~ | No | 57 | Alive | 2020 |
| Jiang et al.[28] | 62 | Male | ~ | Tail | 3 | 1 | 1 | DP | No | Gemcitabine | ~ | ~ | Yes | 3 | Dead | 2021 |
| Cavalcanti et al.[29] | 67 | Male | ~ | Head | 2 | 1 | 0 | PPPD | No | No | ~ | ~ | No | 5 | Alive | 2021 |
| Olayinka et al.[30] | ~ | Male | ~ | Body and tail | 4 | 1 | 0 | DP | No | Gemcitabine+capecitabine | ~ | ~ | No | 3 | Alive | 2021 |
| Rusu et al.[31] | 76 | Female | ~ | Head | 3 | 0 | 0 | PD | No | No | ~ | ~ | No | 4 | Dead | 2021 |
| Smith et al.[32] | 69 | Male | ~ | Head | 3 | 1 | 0 | PPPD | No | Modified FOLFIRINOX | ~ | ~ | No | 3 | Dead | 2021 |
| Aldhaheri et al.[33] 1 | 75 | Male | ~ | Neck | 2 | 0 | 0 | PD | No | No | ~ | ~ | No | 38 | Alive | 2021 |
| Igarashi et al.[34] | 63 | Male | ~ | Head | 4 | 1 | 0 | PD | No | FOLFIRINOX | 20 | ~ | No | 6 | Alive | 2022 |
| Sozutek et al.[35] | 52 | Female | ~ | Head | 3 | 0 | 0 | PD | Yes | Yes | ~ | ~ | No | 20 | Alive | 2022 |
| Sun et al.[36]1 | 56 | Male | ~ | Head | 2 | 0 | 0 | PD | No | Gemcitabine+capecitabine | 6 | ~ | No | 14 | Alive | 2022 |
| Sun et al.[36]2 | 53 | Female | ~ | Body and tail | 2 | 0 | 0 | DP | No | No | ~ | Patient rejects | No | 13 | Alive | 2022 |
| Ota et al.[37] | 76 | Male | ~ | Body and tail | 2 | 0 | 0 | DP | No | Yes | ~ | ~ | No | 21 | Alive | 2022 |
| Masaki et al.[38] | 58 | Female | ~ | Body | 2 | 0 | 0 | DP | Yes | Gemcitabine+S-1 | ~ | ~ | No | 53 | Alive | 2022 |
| Vunk et al.[39] | 67 | Female | ~ | Body and tail | 3 | 0 | 0 | DP | No | No | ~ | ~ | No | 9 | Dead | 2022 |
| Zhao et al.[40] 1 | 71 | Male | ~ | Body and tail | 3 | 1 | 0 | DP | No | No | ~ | ~ | No | 10 | Dead | 2023 |
| Zhao et al.[40] 2 | 65 | Male | ~ | Head | 3 | 1 | 0 | PD | No | No | ~ | ~ | No | 36 | Alive | 2023 |
| Zhao et al.[40] 3 | 46 | Male | ~ | Neck and body | 2 | 0 | 0 | DP | No | No | ~ | ~ | No | 1 | Dead | 2023 |
| Zhao et al.[40] 4 | 37 | Female | ~ | Tail | 3 | 0 | 0 | PD | No | Yes | 6 | ~ | No | 8 | Alive | 2023 |
| Chan et al.[41] | 54 | Male | Asian | Tail | 4 | 0 | 0 | DP | No | FOLFIRINOX+AG | 2 | ~ | No | 11 | Dead | 2023 |
| Lages et al.[42] | 60 | Male | ~ | Head | 2 | 0 | 0 | PD | No | Yes | ~ | ~ | No | 10 | Alive | 2023 |
| Tambasco et al.[43] 1 | 71 | Male | ~ | Body and tail | 3 | 0 | 0 | STP | SBRT | FORFIRI+AG | ~ | ~ | No | 39 | Alive | 2024 |
| Tambasco et al.[43] 2 | 74 | Male | ~ | Head | 2 | 1 | 0 | PD | No | Gemcitabine | ~ | ~ | No | 14 | Dead | 2024 |
| Hrudka et al.[44] 1 | ~ | ~ | ~ | ~ | 2 | 1 | 0 | 1 | No | Yes | ~ | ~ | No | 5.7 | Dead | 2024 |
| Hrudka et al.[44] 2 | ~ | ~ | ~ | ~ | 3 | 0 | 0 | 1 | No | No | ~ | ~ | No | 1.2 | Dead | 2024 |
| Hrudka et al.[44] 4 | ~ | ~ | ~ | ~ | 1 | 0 | 0 | 1 | No | Yes | ~ | ~ | No | 56.4 | Alive | 2024 |
| Hrudka et al.[44] 5 | ~ | ~ | ~ | ~ | 3 | 1 | 0 | 1 | No | Yes | ~ | ~ | No | 58.3 | Alive | 2024 |
| Hrudka et al.[44] 10 | ~ | ~ | ~ | ~ | 2 | 0 | 1 | 1 | Yes | Yes | ~ | ~ | Yes | 171 | Dead | 2024 |
| Hrudka et al.[44] 11 | ~ | ~ | ~ | ~ | 1 | 0 | 0 | 1 | No | No | ~ | ~ | No | 98 | Alive | 2024 |
| Hrudka et al.[44] 12 | ~ | ~ | ~ | ~ | 1 | 0 | 0 | 1 | No | Yes | ~ | ~ | No | 49 | Alive | 2024 |

**Abbreviation: T:** stage Tumor; **N:** stage Node; **M:** stage Metastasis; **PTR (primary tumor resection):** DP: distal pancreatectomy; PD: pancreatoduodenectomy; PPPD: pylorus-preserving pancreaticoduodenectomy; SPDP: spleen-preserving distal pancreatectomy; STP: subtotal pancreatectomy; TP: total pancreatectomy; **Chemotherapy:** AG: albumin paclitaxel + gemcitabine; FORFIRI: leucovorin calcium + fluorouracil + irinotecan; FOLFIRINOX: oxaliplatin + irinotecan + fluorouracil + leucovorin calcium; S-1: an oral anticancer drug which is formulated with tegafur; TS-1: tegafur + gimeracil + oteracil potassium

**Reference**

1. Njoumi N, Elalami FH, Attolou G, et al. Undifferentiated pancreatic carcinoma with osteoclast-like giant cells: a case report. J Gastrointest Cancer. 2014 Dec;45 Suppl 1(Suppl 1):96-8.
2. Jo S. Huge undifferentiated carcinoma of the pancreas with osteoclast-like giant cells. World J Gastroenterol. 2014;20(10):2725-2730.
3. Temesgen WM, Wachtel M, Dissanaike S. Osteoclastic giant cell tumor of the pancreas. Int J Surg Case Rep. 2014;5(4):175-9.
4. Jung H, Kim C, Bae S. Anaplastic carcinoma with osteoclast-like ginat cells of the pancreas.HPB 2014 16 SUPPL. 2 (661-).
5. Kobayashi S, Nakano H, Ooike N, et al.. Long-term survivor of a resected undifferentiated pancreatic carcinoma with osteoclast-like giant cells who underwent a second curative resection: A case report and review of the literature. Oncol Lett. 2014;8(4):14~-1504.
6. Gao HQ, Yang YM, Zhuang Y, et al.. Locally advanced undifferentiated carcinoma with osteoclast-like giant cells of the pancreas. World J Gastroenterol. 2015;21(2):694-698.
7. Chiarelli M, Guttadauro A, Gerosa M, et al.. An indeterminate mucin-producing cystic neoplasm containing an undifferentiated carcinoma with osteoclast-like giant cells: a case report of a rare association of pancreatic tumors. BMC Gastroenterol. 2015;15:161.
8. Sunahara M, Ueki S, Ono Y, et al.. Undifferentiated pancreatic carcinoma with osteoclast-like giant cells showing tumor thrombus into the main pancreatic duct: Report of a case with osteoid formation. Pancreas. 2015 44:8 (1417-).
9. Georgiou GΚ, Balasi E, Siozopoulou V, et al.. Undifferentiated carcinoma of the head of pancreas with osteoclast-like giant cells presenting as a symptomatic cystic mass, following acute pancreatitis: Case report and review of the literature. Int J Surg Case Rep. 2016;19:106-108.
10. Saito H, Kashiyama H, Murohashi T, et al.. Case of Six-Year Disease-Free Survival with Undifferentiated Carcinoma of the Pancreas. Case Rep Gastroenterol. 2016;10(2):472-478.
11. Luchini C, Pea A, Lionheart G, et al.. Pancreatic undifferentiated carcinoma with osteoclast-like giant cells is genetically similar to, but clinically distinct from, conventional ductal adenocarcinoma. J Pathol. 2017 Oct;243(2):148-154.
12. Yazawa T, Watanabe A, Araki K, et al.. Complete resection of a huge pancreatic undifferentiated carcinoma with osteoclast-like giant cells. Int Cancer Conf J. 2017 Aug 1;6(4):193-196.
13. Okamuara K, Ohara M, Kaneko T, et al.. Undifferentiated carcinoma with osteoclast-like giant cells of the pancreas: A case report.Journal of Gastroenterology and Hepatology. 2017 32 Supplement 3 (207-208).
14. Guo YL, Ruan LT, Wang QP, et al.. Undifferentiated carcinoma with osteoclast-like giant cells of pancreas: A case report with review of the computed tomography findings. Medicine (Baltimore). 2018;97(48):e13516.
15. Zhang L, Lee JM, Yoon JH, et al.. Huge and recurrent undifferentiated carcinoma with osteoclast-like giant cells of the pancreas.[In Process] Quantitative Imaging in Medicine and Surgery 2018 8:4 (457-460).
16. Hanayneh W, Parekh H, Fitzpatrick G, et al.. Two Cases of Rare Pancreatic Malignancies. J Pancreat Cancer. 2019;5(1):26-33.
17. Sato K, Urakawa H, Sakamoto K, et al.. Undifferentiated carcinoma of the pancreas with osteoclast-like giant cells showing intraductal growth and intratumoral hemorrhage: MRI features. Radiol Case Rep. 2019;14(10):1283-1287. Published 2019 Aug 14.
18. Matsubayashi H, Kaneko J, Sato J, et al.. Osteoclast-like Giant Cell-type Pancreatic Anaplastic Carcinoma Presenting with a Duodenal Polypoid Lesion. Intern Med. 2019;58(24):3545-3550.
19. Nehmeh WA, Trak-Smayra V, Tarhini A, et al.. A Case Report Presenting an Undifferentiated Pancreatic Carcinoma with Osteoclastic-Like Giant Cells with an Unusual Indolent Course. Am J Case Rep. 2019;20:1750-1754.
20. Shi L, Grooms K, Clanton J. A rare case of pancreatic carcinoma with osteoclast-like giant cells reported in a young female. HPB 2019 21 Supplement 1 (S118-).
21. Uemura S, Maeda H, Tsujii S, et al.. Anaplastic pancreatic carcinoma growing within the main pancreatic duct complicated by a large pseudocyst.Annals of Cancer Research and Therapy 2019 27:2 (95-100).
22. Miyagawa Y, Kitazawa M, Kitahara H, et al.. Three Curative Pancreatectomies for the Metachronous Appearance of Pancreatic Invasive Ductal Adenocarcinoma. Case Rep Oncol. 2020;13(1):392-397.
23. Yang G, Yin J, Ou K, et al.. Undifferentiated carcinoma with osteoclast-like giant cells of the pancreas harboring KRAS and BRCA mutations: case report and whole exome sequencing analysis. BMC Gastroenterol. 2020 Jun 26;20(1):202.
24. Obayashi M, Shibasaki Y, Koakutsu T, et al.. Pancreatic undifferentiated carcinoma with osteoclast-like giant cells curatively resected after pembrolizumab therapy for lung metastases: a case report. BMC Gastroenterol. 2020 Jul 11;20(1):220.
25. Mattiolo P, Fiadone G, Paolino G, et al.. Epithelial-mesenchymal transition in undifferentiated carcinoma of the pancreas with and without osteoclast-like giant cells. Virchows Arch. 2021 Feb;478(2):319-326.
26. Cai Y, Chen Y, Wu X, et al.. Seven-year disease-free survival in a patient with osteoclast-like giant cell-containing pancreatic undifferentiated carcinoma: a case report and literature review. Int J Clin Exp Pathol. 2020 Dec 1;13(12):3200-3205.
27. Yamamura M, Nebiki H, Sakata Y, et al.. A case of preoperative diagnosis of anaplastic pancreatic cancer with osteoclast-like giant cells which was classified as ts1 (Diameter, 13mm) using endoscopic ultrasound-fine needle aspiration. Gastroenterological Endoscopy 2020 62:9 (1600-1606).
28. Jiang J, Luo J. Osteoclast-like giant cell undifferentiated carcinoma of the pancreas: a case report. Int J Clin Exp Pathol. 2021 Feb 1;14(2):179-185.
29. Cavalcanti E, Schena N, Serino G, et al.. Assessment and management of undifferentiated carcinoma with osteoclastic like giant cells of the pancreas: a case report and revision of literature. BMC Gastroenterol. 2021 Jun 2;21(1):247.
30. Olayinka O, Kaur G, Gupta G. Undifferentiated Pancreatic Carcinoma With Osteoclast-Like Giant Cells and Associated Ductal Adenocarcinoma With Focal Signet-Ring Features. Cureus. 2021 May 12;13(5):e14988.
31. Rusu A, Giuşcă SE, Apostol DGC, et al.. Cephalic undifferentiated carcinoma with osteoclast-like giant cells arising from the main pancreatic duct: case report and literature review. Arch Clin Cases. 2021 Oct 27;6(1):6-21.
32. Smith JL, Jacovides CL, Tucker CM, et al.. Sequencing of an Undifferentiated Carcinoma with Osteoclast-Like Giant Cells of the Pancreas: A Case Report. J Pancreat Cancer. 2021 Oct 7;7(1):71-73.
33. Aldhaheri R, Barat M, Dohan A, et al.. Imaging features of undifferentiated carcinoma with osteoclastic giant cells of the pancreas. Diagnostic and Interventional Imaging 2020 101:12 (839-841).
34. Igarashi Y, Gocho T, Taniai T, et al.. Conversion surgery for undifferentiated carcinoma with osteoclast-like giant cells of the pancreas: a case report. Surg Case Rep. 2022;8(1):42.
35. Sozutek A, Elife A. Borderline-resectable Undifferentiated Carcinoma with Osteoclast-like Giant Cells of the Pancreas: upfront Surgery or Neoadjuvant Chemotherapy. Journal of the College of Physicians and Surgeons--Pakistan : JCPSP vol. 32,7 (2022): 934-937.
36. Sun WJ, Chang Q, Zhang TM, et al. [Undifferentiated carcinoma of the pancreas with osteoclast-like giant cells: report of two cases]. Zhonghua Bing Li Xue Za Zhi. 2022 Aug 8;51(8):776-778.
37. Ota K, Abue M, Meguro R, et al.. [A case of anaplastic pancreatic cancer with osteoclast-like giant cells during follow-up of a branch-duct pancreatic intraductal papillary mucinous tumor]. Nihon Shokakibyo Gakkai Zasshi. 2022;119(10):961-968.
38. Masaki K, Makoto F, Ayaka I, et al.. A case report of anaplastic carcinoma with osteoclast-like giant cells arising in the pancreatic body. Journal of Surgical Case Reports, Volume 2022, Issue 6, June 2022, rjac288.
39. Vnuk K, Pavi I, Brleti D, et al.. UNDIFFERENTIATED CARCINOMA OF THE PANCREAS WITH OSTEOCLAST-LIKE GIANT CELLS: REPORT OF TWO CASES. Libri Oncologici 2022 50:1 (39-43).
40. Zhao N, Mei N, Yi Y, et al.. Case report: Pathological and genetic features of pancreatic undifferentiated carcinoma with osteoclast-like giant cells. Pathol Oncol Res. 2023 Mar 3;29:1610983.
41. Chan W, Park S, Shirkhoda L, et al.. Undifferentiated carcinoma of the pancreas with osteoclast-like giant cells: a case report. J Med Case Rep. 2023 Nov 16;17(1):477.
42. Lages Dos Santos J, Sanches A. Undifferentiated carcinoma of the pancreas with osteoclast-like giant cells presenting cyto-histopathologic correlation: A case report. Virchows Archiv 2023 483 (S225-S226) Supplement 1.
43. Tambasco ML, Echelard P, Perrault F, et al.. Undifferentiated carcinoma of the pancreas with osteoclast-like giant cells, a two cases report. Int J Surg Case Rep. 2024 Mar;116:109419.
44. Hrudka J, Kalinová M, Ciprová V, et al.. Undifferentiated Carcinoma with Osteoclast-like Giant Cells of the Pancreas: Molecular Genetic Analysis of 13 Cases. Int J Mol Sci. 2024 Mar 14;25(6):3285.
